# Reduction of inter-observer variability using MRI and CT fusion in delineating of primary tumor for radiotherapy in lung cancer with atelectasis

**DOI:** 10.3389/fonc.2022.841771

**Published:** 2022-08-03

**Authors:** Hongjiao Zhang, Chengrui Fu, Min Fan, Liyong Lu, Yiru Chen, Chengxin Liu, Hongfu Sun, Qian Zhao, Dan Han, Baosheng Li, Wei Huang

**Affiliations:** ^1^ Shandong Cancer Hospital and Institute, Shandong First Medical University and Shandong Academy of Medical Sciences, Jinan, China; ^2^ Department of Radiation Oncology, Shandong Cancer Hospital and Institute, Shandong First Medical University and Shandong Academy of Medical Sciences, Jinan, China; ^3^ West China School of Public Health, Sichuan University, Chengdu, China

**Keywords:** lung neoplasms, pulmonary atelectasis, radiotherapy, magnetic resonance imaging, computed tomography

## Abstract

**Purpose:**

To compare the difference between magnetic resonance imaging (MRI) and computed tomography (CT) in delineating the target area of lung cancer with atelectasis.

**Method:**

A retrospective analysis was performed on 15 patients with lung cancer accompanied by atelectasis. All positioning images were transferred to Eclipse treatment planning systems (TPSs). Six MRI sequences (T1WI, T1WI+C, T1WI+C Delay, T1WI+C 10 minutes, T2WI, DWI) were registered with positioning CT. Five radiation oncologists delineated the tumor boundary to obtain the gross tumor volume (GTV). Conformity index (CI) and dice coefficient (DC) were used to measure differences among observers.

**Results:**

The differences in delineation mean volumes, CI, and DC among CT and MRIs were significant. Multiple comparisons were made between MRI sequences and CT. Among them, DWI, T2WI, and T1WI+C 10 minutes sequences were statistically significant with CT in mean volumes, DC, and CI. The mean volume of DWI, T2WI, and T1WI+C 10 minutes sequence in the target area is significantly smaller than that on the CT sequence, but the consistency is higher than that of CT sequences.

**Conclusions:**

The recognition of atelectasis by MRI was better than that by CT, which could reduce interobserver variability of primary tumor delineation in lung cancer with atelectasis. Among them, DWI, T2WI, T1WI+C 10 minutes may be a better choice to improve the GTV delineation of lung cancer patients with atelectasis.

## Introduction

For both men and women, lung cancer is the most common diagnosis of cancer and the leading cause of cancer deaths (1). Radiotherapy is an important treatment for patients with lung cancer, especially for patients with advanced unresectable lung cancer (2). The target description of lung cancer has great variability, especially for lung cancer patients with atelectasis (3–5). The variability of the contour structure may lead to insufficient doses, lower tumor control probability, or excessive doses, resulting in an increase in the probability of normal tissue complication (NTCP). The ability of CT to recognize soft tissue is so limited that it cannot distinguish tumor from atelectasis. Many scholars have begun to explore other imaging methods to distinguish tumors from atelectasis, such as PET-CT and MRI. Related studies have proved that compared with PET-CT, MRI has better spatial resolution and contrast between normal and cancerous tissues (6–9). It is more accurate and repeatable in delineating the target area of lung cancer (10).

However, there is no in-depth study to explore the effect of different MRI sequences on the interobserver difference of target delineation in lung cancer with atelectasis. The purpose of this study is to explore the differences between different MRI-CT fusion sequences and single CT, so as to select the optimal MRI sequence for GTV target delineation of lung cancer with atelectasis.

## Materials and methods

### Cohort of patients

We retrospectively included 15 lung cancer patients who underwent positioning CT and MRI scans between May 2019 and June 2021. Due to the small number of lung cancer patients who underwent MRI localization, only 15 patients who met the criteria for enrollment were included during the study period. The 15 patients recruited in the study were pathologically diagnosed with lung cancer and imaging diagnosed with atelectasis. The diagnosis of atelectasis was decided by a senior doctor in the imaging department. CT- and MRI-simulated localization images were scanned in the same supine position within a week. Among them, six MRI sequences (T1WI, T1WI+C, T1WI+C Delay, T1WI+C 10 minutes, T2WI, DWI) were all scanned completely during MRI location scanning. Patients with surgically removed or metastatic lung tumors, unclear tumor display, contraindications to MRI, or intolerance were excluded. All the 15 patients in the group were diagnosed with central lung cancer. Among them, one patient had not received treatment, 11 patients had received chemotherapy, one patient had received chemotherapy and immunotherapy, one patient had received chemotherapy and anti-angiogenesis therapy, and one patient had received chemotherapy, immunity, and anti-angiogenesis therapy. The detailed data of the patients are listed in **Table 1**.

**Table 1:** Clinical characteristics of the 15 included patients.

Patient characters	No. of patients	%
Gender		
Male	11	73.3
Female	4	26.7
Age		
Median (year)	64.87	
50-60	3	20
60-70	9	60
70-80	3	20
Location		
Upper lobe of left lung	7	46.7
lower lobe of left lung	1	6.7
Left hilum of lung	2	13.3
Upper lobe of right lung	2	13.3
lower lobe of right lung	2	13.3
Middle lobe of right lung	1	6.7
Histology		
Squamous cell carcinoma	6	40
Adenocarcinoma	3	20
Small cell lung cancer	5	33.3
Mucoepidermoid carcinoma	1	6.7
Stage		
III	10	66.7
IV	5	33.7
KPS^†^		
80	8	53.3
90	7	46.7
Mean interval time from positioning CT to MRI (day)	2	

^†^Karnofsky performance status.

### Imaging modalities

#### CT scan and imaging acquisition

All patients underwent CT simulation (Brilliance CT Big Bore; Philips Healthcare, DA Best) before radiotherapy. All of them were fixed with a vacuum negative pressure bag in supine position. Respiratory gating was used during the scan. The CT scan area covered at least the chest. The interslice thickness of the CT scan was 3 mm. At the end of the scan, the enhanced scan was performed by injecting 90 ml iohexol (350 mg/ml).

#### MRI scan and imaging acquisition

The supine position of MRI was the same as the simulated position of CT. A 3.0-T, 70-cm Bore MR scanner (750W, General Electric Co.) was used for MRI simulation positioning in our hospital. A 20-ml high-pressure injector was used to inject gadoterate meglumine (0.2 mmol/kg) at 2 ml/s followed by 20 ml saline to obtain the enhanced image. Meglumine gadolinate is a paramagnetic contrast agent. A low-dose injection only shortens the T1WI relaxation time and does not affect T2WI imaging. The MRI scanning sequence includes T1WI (WATER : BH AX LAVA-Flex), T1WI+C (WATER : BH AX LAVA-Flex-C), T1WI+C Delay (WATER : BH AX LAVA-Flex-C delay), T1WI+C 10 minutes (WATER : BH AX LAVA-Flex-C 10min), T2WI (RTr Ax T2 fs Propeller), and DWI, in which T1WI+C 10 minutes was specially scanned by our organization. T1WI+C, T1WI+C Delay, and T1WI+C 10 minutes sequences were scanned 15 s, 2 min, and 10 min after injection of a contrast medium, respectively. Because of the large endothelial space and immaturity of the vascular endothelium in the tumor, the contrast medium enters and exits faster than the normal tissue. During the early delayed imaging scan, the contrast medium enters the tumor tissue, and the normal lung tissue begins to strengthen, but the enhancement is not complete. At this time, the boundary between tumor and normal tissue is not clear. When scanning on the 10-min image, the normal lung tissue was completely enhanced, while the tumor tissue showed fast-in and fast-out realization. At this time, all the contrast media had been leaked, and the low signal of the tumor tissue was more obvious compared with that of the fibrous tissue and normal tissue, and the tumor boundary was clearer. We wonder whether T1WI+C 10 minutes sequences can also be used to distinguish lung cancer from atelectasis. This sequence has been proved to be helpful in the delineation of breast cancer lumpectomy cavity (11). The specific principle of the T1WI+C 10 minutes sequence needs to be further studied. The sequence of MRI scanning was T1WI, T1WI+C, T1WI+C Delay, DWI, T2WI, and T1WI+C 10 minutes. In addition, we used respiratory gating in MRI scans to reduce MRI motion artifacts. The patient needs to hold his breath for 18 s during each T1-enhanced correlation sequence scan of the chest. Respiratory trigger was used in T2WI and DWI sequence scanning. The slice thickness of all MRI sequences except DWI was 3 mm. The slice thickness of the DWI scan was 3.6 mm, and there was no scanning interval. In order to keep the consistency of the layer thickness, the DWI image was reconstructed to the 3-mm layer thickness. The repetition time (TR), echo time (TE), and matrix of T1WI, T1WI+C, T1WI+C Delay, and T1WI+C 10 minutes were 4.2 ms, 1.7 ms, 280 × 192, and the field of view (FOV) was 44 × 44, 44 × 44, 44 × 35.2, and 44 × 35.2, respectively. The TR, TE, and matrix of T2W were 7,059 ms, 79 ms, and 384 × 384, respectively. The DWI sequence TE was 60 ms, and the matrix was 128 × 128. The B value was 600 s/mm^2^.

### Imaging fusion

We used respiratory gating in CT and MRI positioning scans to reduce motion artifacts. The analog positioning images of CT and MRI were uploaded to Eclipse treatment planning systems (TPSs). According to the anatomy of the sternum, rib, vertebral body, pulmonary artery, and tumor, each MRI sequence (T1WI, T1WI+C, T1WI+C Delay, T1WI+C 10 minutes, T2WI, DWI) was automatically registered with CT through the planning system, and the fusion stability was increased by manual correction.

### Structure delineation

**Figure 1:**
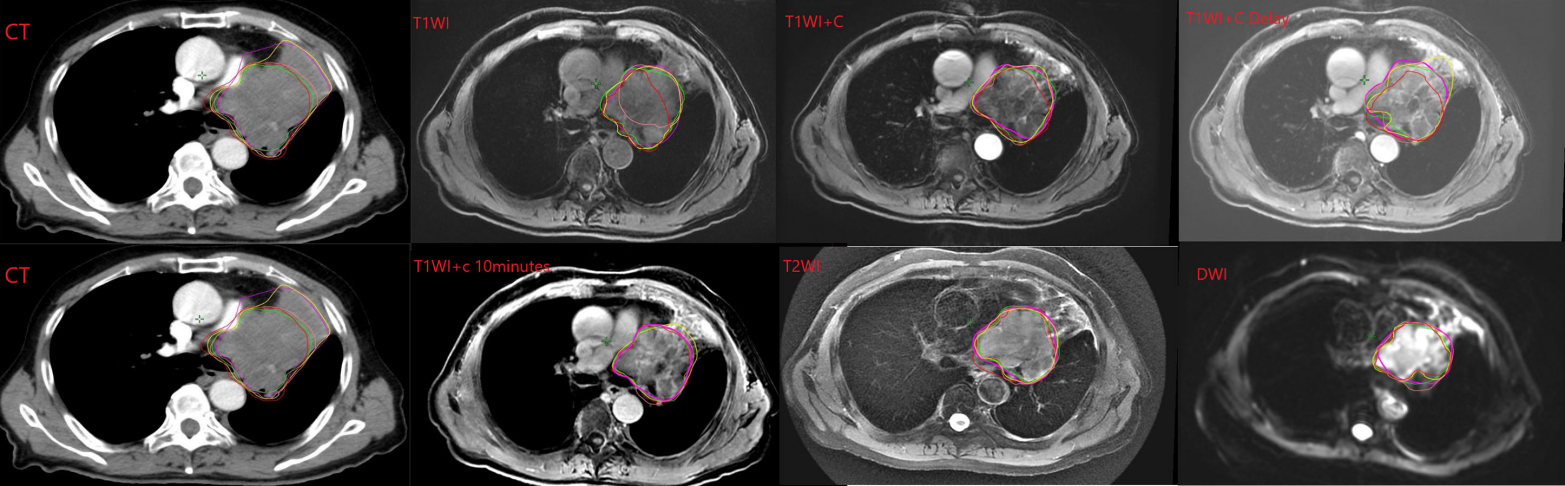
Example of GTV contoured on the different CT and MRI sequences by each observer. Note: The lines of different colors in the picture represent different observers.


Five radiation oncologists come from our hospital with 17, 16, 14, 7, and 6 years of working experience, respectively. The five observers, all of whom have rich clinical experience and are good at target delineation radiotherapy, were chosen in order to reduce the intra-observer variability. This paper mainly discusses the differences between observations. The five radiation oncologists sketched the tumor contours on CT and CT-MRI fusion images, respectively. The boundary between tumor and atelectasis was determined by referring to the CT structure and coronal and sagittal MRI images when drawing CT-MRI fusion images. The target areas of CT and MRI-CT images were sketched on the same day. We stipulate that CT images are drawn first. Then, CT-MRI image fusion was performed. The fusion image was drawn in the order of T1WI, T1WI+C, T1WI+C Delay, T1WI+C 10 minutes, T2WI, and DWI sequences. According to previous reports, observers were trained in advance to outline only the primary tumor and not the metastatic lymph nodes (12–14). If the lymph node is fused with the primary tumor, it can be delineated (see **Figure 1**). Prior to the study, all observers had no access to the medical records of all patients and the contours of other observers. In the course of the study, all observers delineated the target independently and were not affected by other factors. The primary tumor that has been delineated in the target area is defined as the standard total target volume (GTV). In this article, the mean volume refers to the average volume delineated by the five observers. Conformity index (CI) and Dice coefficient (DC) were used in articles with similar research methods, which can be used to express the consistency of delineation between observations (15). The mean volume, CI, and DC of each sequence were compared to determine the variability between observers. Related studies have shown that the difference in GTV between different images is due to the shape of the target outline rather than the position of the target center (10). Therefore, in this paper, the volume of the target area is compared, but the center position of the target area is not compared separately. The values of DC and CI also reflected the overlap of the delineator’s target area. CI was equal to the ratio of the overlapping volume to the common volume of all observers. The value of DC was calculated using the ratio of overlapping volume and the average volume contoured by the five observers. The higher the CI and DC values, the higher the consistency of the five observers’ delineation in the target area of a certain sequence, and the smaller the difference. The specific formula of the parameter is as follows:

$$\text{CI}=\frac{\text{A}\cap^{}\text{B}\cap^{}\text{C}\cap^{}\text{D}\cap^{}\text{E}}{\text{A}\cup^{}\text{B}\cup^{}\text{C}\cup^{}\text{D}\cup^{}\text{E}}\text{ DC}=\frac{5\left(\text{A}\cap^{}\text{B}\cap^{}\text{C}\cap^{}\text{D}\cap^{}\text{E}\right)}{\text{A}+\text{B}+\text{C}+\text{D}+\text{E}}$$

### Statistical analysis

**Figure 2:**
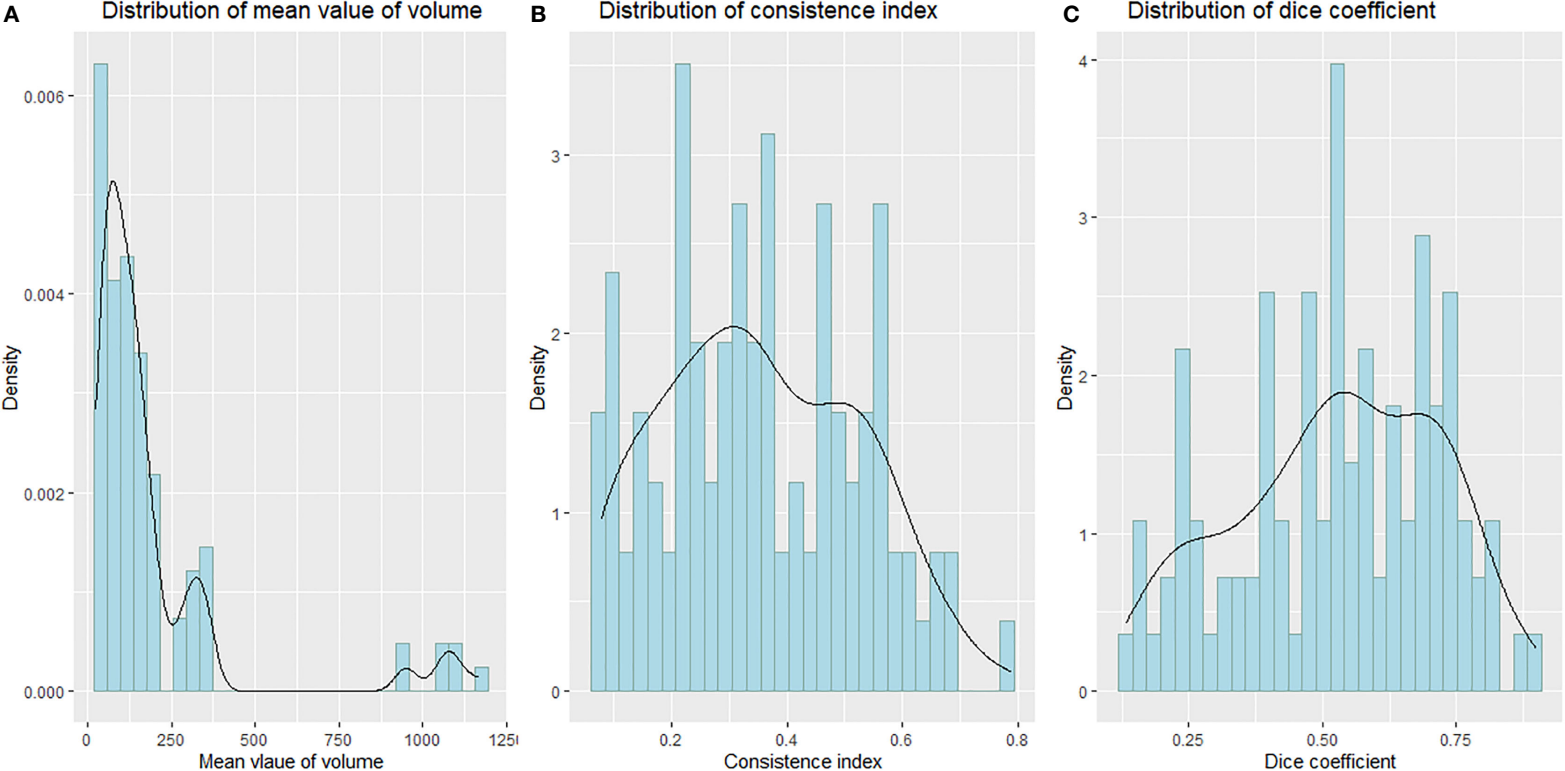
Probability density distribution curve histogram of the mean volume, CI, and DC.

All analyses were conducted using R software (version 4.0.2) and SPSS software (Version 25.0). *P*< 0.05 was used to determine statistical significance. The Shapiro–Wilk test was used to test the normality of the distribution of tumor volume, CI, and DC, respectively (16). The W values of the Shapiro–Wilk test for tumor volume, CI, and DC distribution were 0.57218 (*P*< *0.01*), 0.97213 (*P = 0.02592*), and 0.96955 (*P = 0.01611*), respectively, suggesting that the distribution of tumor volume, DC, and CI is not normal, as shown in **Figure 2**. We used Friedman M test to compare the differences of different sequences in CI, DC, and tumor volume. The Wilcoxon rank-sum test was used to compare different MRI sequences with CT pairwise. The Bonferroni method was used to account for multiple comparisons; the corrected P-value was 0.0083. A *P-value <0.0083* was considered to be statistically significant.

## Results

### Patient’ delineation volume comparison

The median and quartile spacing of the volumes of CT and MRI sequences as well as the results of statistical analysis are shown in **Table 2**. The average GTV volume drawn by the five observers was significantly different between the fusion sequences of CT-MRI and CT alone (*P<0.01*).

**Table 2:** Descriptive results of mean volumes, CI, and DC and results of Friedman test.

Sequence	Consistency index	Dice coefficient	Volume
CT	0.28 (0.19~0.48)	0.47 (0.33~0.69)	139.90 (83.05~200.70)
T1WI	0.25 (0.14~0.36)	0.46 (0.26~0.56)	116.60 (86.95~190.30)
T1WI+C	0.24 (0.17~0.39)	0.47 (0.31~0.61)	112.70 (70.70~204.65)
T1WI+C Delay	0.29 (0.22~0.35)	0.48 (0.38~0.59)	127.60 (67.90~182.00)
T1WI+C 10 minutes	0.40 (0.30~0.53)	0.60 (0.52~0.73)	101.40 (64.05~163.95)
T2WI	0.47 (0.34~0.55)	0.68 (0.52~0.74)	105.50 (59.30~148.10)
DWI	0.42 (0.34~0.58)	0.55 (0.53~0.74)	107.00 (54.30~148.45)
M	43.478	34.79	35.764
P	<0.001	<0.001	<0.001

† The numbers in parentheses represent quartile spacing. The numbers outside the parentheses represent the median.

The six MRI sequences were compared with CT sequences in pairs. The results showed that there were statistical differences in the T1WI+C 10 minutes, T2WI, and DWI sequences compared with the CT sequences (P = 0.002, 0.002, 0.002 < 0.0083). As seen in **Table 2**, the median of the CT sequence was 139.9, while the median of the T1WI+C 10 minutes, T2WI, and DWI sequences was 101.40, 105.50, and 107.00, all less than that by CT. This shows that the volume sketched by T1WI+C 10 minutes, T2WI, and DWI sequences is smaller than that delineated by CT.

### Consistency of GTVs

**Figure 3:**
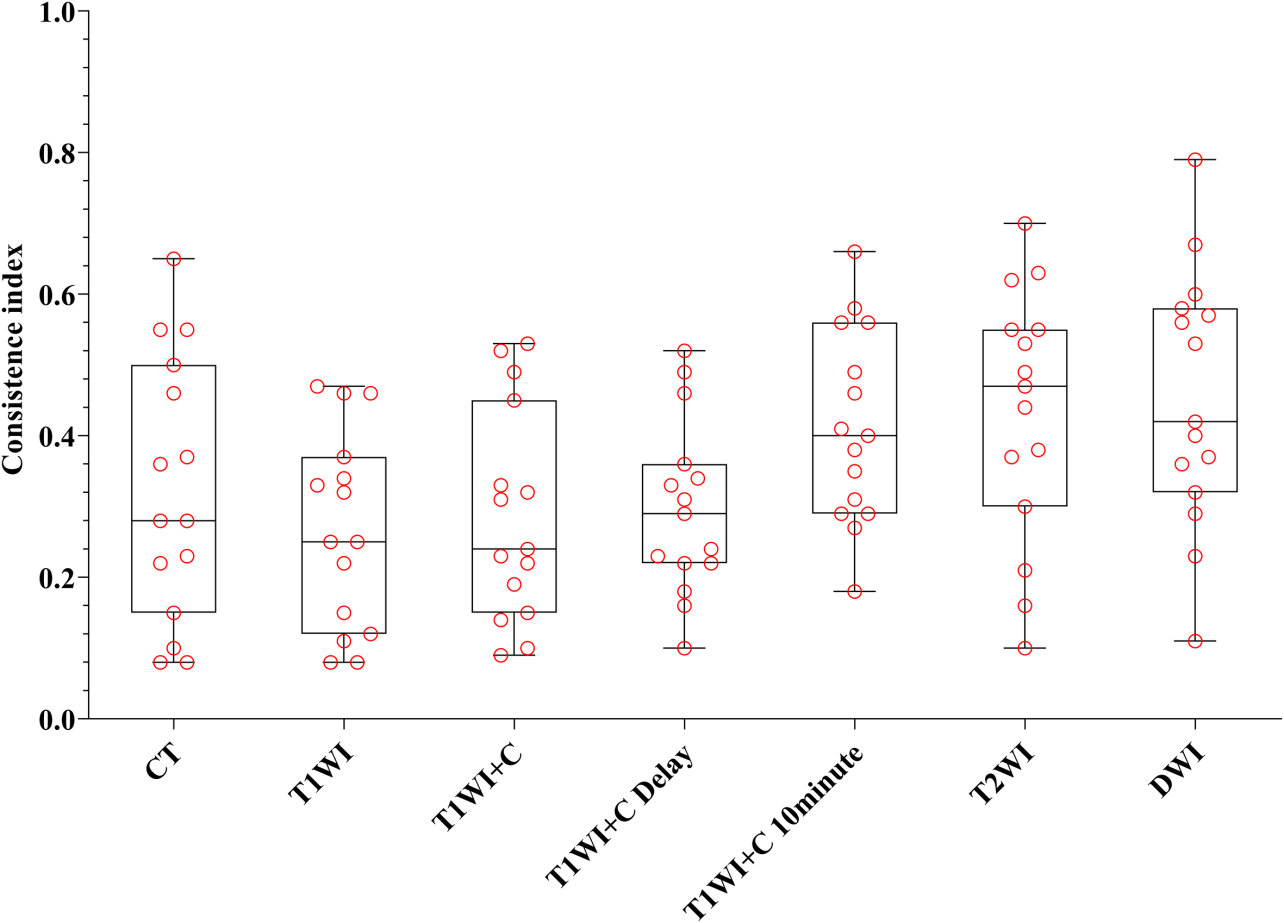
The boxplot of CI. The black line of the box is the median, and the length of the box represents the interquartile range.

**Figure 4:**
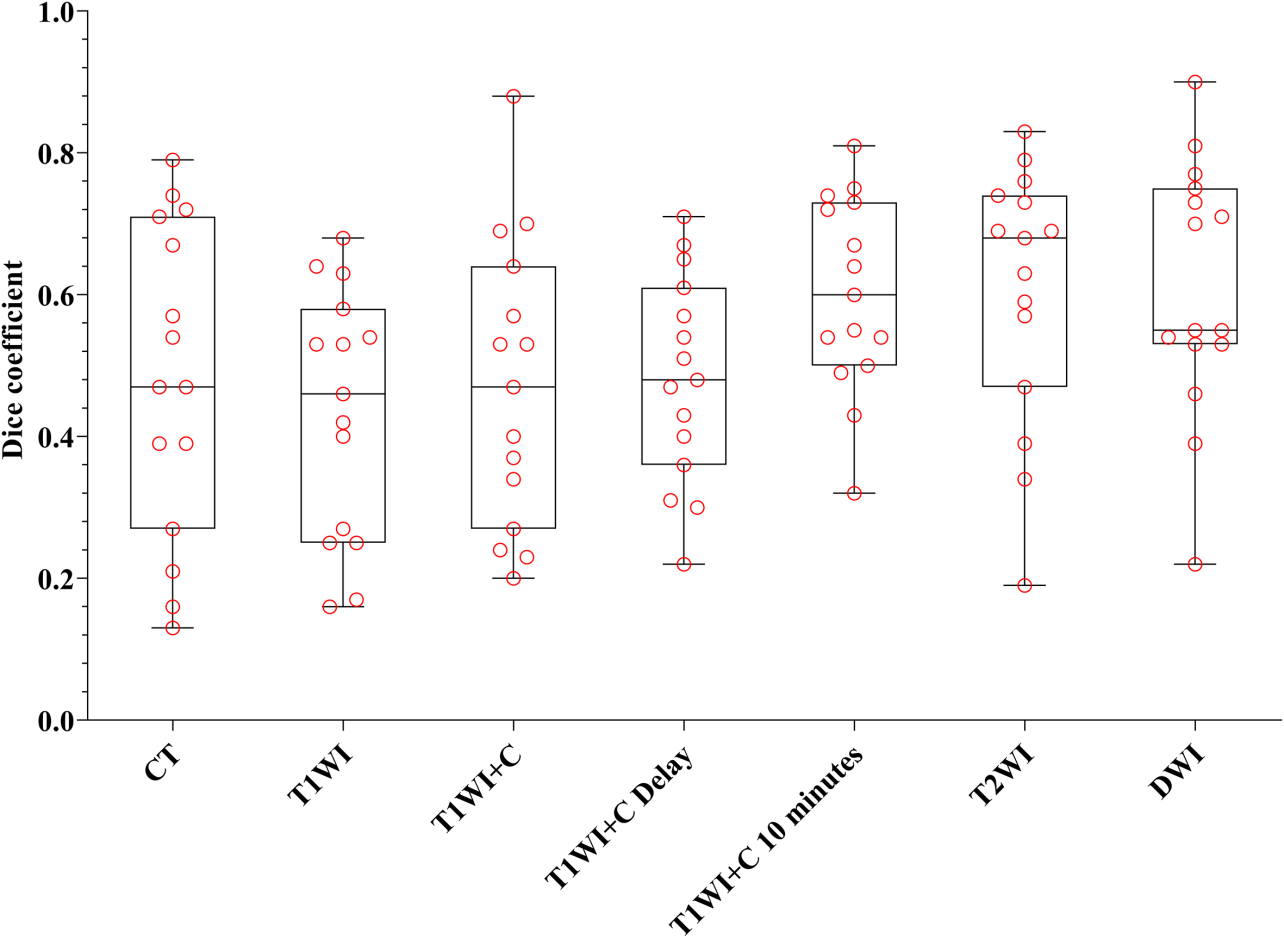
The boxplot of DC. The black line of the box is the median, and the length of the box represents the interquartile range.

Every descriptive statistic of CI and DC is also found in **Table 2**. We can see from **Figures 3** and **4** the box diagrams of CI and DC; it can be seen that the median value and variability of the DWI, T2WI, and T1WI+C 10-minute sequence are better than those of CT.

**Table 2** shows that there were statistical differences in consistency index and DC between CT and MRI sequences. Taking CT as a reference, six MRI sequences were compared with CT sequences in pairs. It is seen in **Table 3** that the CI and DC values of the T1WI + C 10 minutes, T2WI, and DWI sequences were statistically different from those of the CT sequence. As mentioned above, it is seen in **Figures 3** and **4** that the median of the three sequences is larger than that of CT. It can be concluded that the CI and DC values of the T1WI+C 10 minutes, T2WI, and DWI sequences are greater than those of the CT sequences.

**Table 3:** The results of the Wilcoxon rank-sum test of mean volumes, CI, and DC.

Sequence	Consistency index		Dice coefficient		Volume	
	Z	P	Z	P	Z	P
CT *vs*. T1WI	1.364	0.172	0.995	0.32	1.136	0.256
CT *vs*. T1WI+C	0.881	0.378	0.369	0.712	1.695	0.09
CT *vs*. T1WI+C Delay	0.37	0.712	0.251	0.802	1.533	0.125
CT *vs*. T1WI+C 10 minutes	2.849	0.004*	2.758	0.006*	3.124	0.002*
CT *vs*. T2WI	3.112	0.002*	3.079	0.002*	3.124	0.002*
CT *vs*. DWI	3.239	0.001*	3.015	0.003*	3.124	0.002*

* p< 0.0083.

## Discussion

In this study, we compared the differences of target volume, CI, and DC in locating MRI and CT in patients with lung cancer with atelectasis. DWI, T2WI, and T1WI+C 10 minutes sequences were statistically significant with CT in volume, CI, and DC. We can conclude that contours of the observers on CT overestimate the GTV, while MRI, especially the DWI, T2WI, and T1WI+C 10 minutes sequences, can better delineate the target area of lung cancer with atelectasis.

GTV definition in lung cancer is one of the cornerstones in quality assurance of radiotherapy (17, 18). However, there is no unified target delineation standard for lung cancer patients with atelectasis. Contouring variability is significantly larger on the atelectasis interface for all modalities compared with other interfaces (19). Due to its poor soft resolution, CT alone may lead to large differences between observers or within observers. Therefore, many radiation oncologists use a variety of measures to improve the differentiation between tumors and atelectasis.

Many related studies have shown that MRI has a certain advantage in distinguishing atelectasis from tumor tissue because of its good soft tissue recognition ability (19–21). Among them, T2WI and DWI sequences have been proved to be helpful in the differentiation of lung cancer from atelectasis. As technology advances, radiation oncologists begin to experiment with image fusion to increase the accuracy of target delineation and reduce differences between observers. Steenbakkers et al. (18) believed that multimodal imaging and combining different imaging features may be the best way to define GTV most accurately. Karki et al.’s research also confirmed this view (22). According to the study of Fleckenstein et al. (23), the GTV sketched by DWI is much larger than the GTV sketched by PET-CT in non-small cell lung cancer. The safety and effectiveness of DWI-based radiotherapy planning need to be further verified by prospective studies. However, Basson et al. (24) have compared the GTV of lung cancer lesions on CT, MRI, and PET images. The results show that compared with PET, MRI seems to reduce the interobserver variability of GTV depiction of ill-defined lung tumors. MRI can be used to differentiate tumors from atelectasis.

The study by Zhang et al. (10) compared the distance between the centroids of CT, PET-CT, and MRI, and there was no significant difference. This showed that differences in GTVs using different procedures are mainly attributable to the shape of the target delineation, but not the location of the target centers. On the one hand, many studies have confirmed that PET-CT and MRI are better than CT in differentiating tumor from atelectasis. On the other hand, related studies show that DWI-MRI has the highest target delineation accuracy and the lowest variation compared with PET-CT. Therefore, we believe that compared with FDG-PET, MRI may become a more general and practical tool for treatment planning (25). At present, many studies focus on the DWI sequence, but few studies discuss the role of other MRI sequences in the target delineation of lung cancer patients with atelectasis.

Six MRI sequences and not only DWI were studied in this study. Moreover, the effects of T1WI enhancement sequences on each time image were studied from pulmonary vascular perfusion imaging. At present, there is no literature to study whether MRI sequence injection contrast medium 10 minutes scan images can help distinguish tumor and atelectasis. In addition, few articles use CI and DC values to further quantify the differences between interobservers. This is the innovation point of this article.

Studies by Weiss et al. (26) suggest that the lack of continuous education and training is one of the reasons for the variability of tumor contours. Bowden et al. (27) found that the application of the delineation protocol can improve the accuracy of sketching. The average variation rate of the measured GTV decreased from 20% without a plan to 13% with a plan. In clinical practice, all observers have little experience in sketching GTV on MRI. Research by Konert et al. (28) shows that multiple training interventions improve PET/CT-based based target volume delineation accuracy in NSCLC and reduce interobserver variation. In this study, the observers have trained uniformly before contouring the target; only the focus area of the primary tumor was delineated, not the lymph nodes. If the focus of the tumor fuses with the lymph nodes, they are delineated. The error caused by metastatic lymph nodes is reduced. In addition, in treatment planning, positioning technology such as MRI has advanced, whose superior soft-tissue contrast can reduce the uncertainty of organ description (29).

The overall trend shows that the T1WI+C 10 minutes, T2WI, and DWI sequences are obviously superior to those of CT in target delineation, which may help improve the consistency of target delineation in patients with atelectasis of lung cancer and reduce the target volume and normal tissue volume and be better used in clinical practice. In recent years, MRI has been increasingly used in treatment planning in radiotherapy. With the advent of the era of precision radiotherapy, the definition of the tumor target volume is particularly important for conformal intensity-modulated radiotherapy. Compared with CT, MRI can provide better soft-tissue features, coupled with its multi-plane ability and enhanced imaging function; these advantages for target volume description outweigh its lack of electronic density information and potential image distortion. MRI radiation-less imaging, high time resolution, fast sequence, and functional imaging highlight the potential of MRI to improve accuracy. MRI-based delineation provides a better target description for radiotherapy treatment planning (RTP). Furthermore, MRI not only is suitable for initial radiotherapy of tumors but also may be used for retreatment, because it can distinguish between changes caused by cancer recurrence and changes caused by fibrosis after treatment. It can also better depict risk organs (OAR) to avoid doses in RTP (30–32). At present, texture analysis, automatic delineation, 4D-MRI, MRI accelerator, and other emerging technologies are gradually applied in clinical settings (33–36). This study may provide a new application direction for radiotherapy guided by MRI accelerator in the future.

In the course of radiotherapy, the regression or progression of the tumor can lead to the regression or expansion of atelectasis. Atelectasis changes in 10% to 30% of patients with non-small cell lung cancer during treatment (37). Tennyson et al. (38) also showed that during radiotherapy, atelectasis and primary tumor volumes decreased on average 136.7 cm^3^ (20–369 cm^3^) for atelectasis and 40 cm^3^ (-7 to 131 cm^3^) for primary tumor. It is very important to detect the changes in atelectasis volume during treatment. In practical clinical work, lung cancer patients with atelectasis will be scanned by CBCT imaging every time they are treated with radiotherapy to closely observe the changes of atelectasis, modify the target area in time, and reduce the dose of normal tissue. At present, there is no related research on predicting the geometric changes of lung tissue caused by radiotherapy for atelectasis of lung cancer, which may be a direction of future research.

This study does contain some limitations. Firstly, due to the strict inclusion criteria, the sample size is significantly reduced. Our team is now expanding the sample size for follow-up automatic sketching-related research. Secondly, target delineation lacks the gold standard of pathology, which may need to be further confirmed by prospective studies. Thirdly, the imaging findings of atelectasis and obstructive pneumonia are different in different periods. In the process of delineating, the inflammation period cannot be determined, which undoubtedly increases the difficulty of delineating the target area, which may increase the difference between the observers. Fourthly, the limitation of rigid registration itself could result in inevitable errors, which increases the contingency of the results. Fifthly, PET-CT could have been another modality studied.In addition, there is inevitably some recall bias. Finally, heterogeneity of the study group may affect the results.

In summary, compared with CT, MRI may help to distinguish tumor from atelectasis. In particular, compared with other MRI sequences, T1WI+C 10 minutes is a new discovery. The results of T2WI and DWI are consistent with those of other studies, and they have good identification ability. The formulation of the MRI and CT dual-localization radiotherapy plan may be helpful to reduce the target volume and normal-tissue dose in patients with lung cancer with atelectasis. This study may provide a new direction for MRI-guided radiotherapy. In addition, the prediction of geometric changes of the target area before treatment in patients with lung cancer atelectasis is also worthy of further study.

## Data availability statement

The original contributions presented in the study are included in the article/supplementary material. Further inquiries can be directed to the corresponding author.

## Ethics statement

The studies involving human participants were reviewed and approved by ethics committee of Shandong Cancer Hospital. Written informed consent for participation was not required for this study in accordance with the national legislation and the institutional requirements.

## Author contributions

HZ is responsible for the research design, planning implementation, statistical analysis, and drafting of the manuscript. CF, MF, LL, YC, HS, QZ, DH, and CL participated in the study design and data acquisition. MF and LL carried out the literature search and data aggregation. BL provided the theoretical proof and academic advice. WH is responsible for the topic selection, overall research guidance, and revision of the paper. All authors contributed to the article and approved the submitted version.

## Funding

This work was supported by the National Natural Science Foundation of China (No. 81773232), Academic Promotion Program of Shandong First Medical University (Shandong Academy of Medical Sciences) (No. 2020RC002), and Project of Young Taishan Scholars (No. Tsqn201909187).

## Conflict of interest

The authors declare that the research was conducted in the absence of any commercial or financial relationships that could be construed as a potential conflict of interest.

## Publisher’s note

All claims expressed in this article are solely those of the authors and do not necessarily represent those of their affiliated organizations, or those of the publisher, the editors and the reviewers. Any product that may be evaluated in this article, or claim that may be made by its manufacturer, is not guaranteed or endorsed by the publisher.

## Abbreviations

CT, computed tomography; GTV, gross tumor volume; MRI, magnetic resonance imaging; TE, echo time; TR, repetition time; T1WI, WATER : BH AX LAVA-Flex; T1WI+C, WATER : BH AX LAVA-Flex-C; T1WI+C Delay, WATER : BH AX LAVA-Flex-C delay; T1WI+C 10 minutes, WATER : BH AX LAVA-Flex-C 10min; T2WI, RTr Ax T2 fs Propeller; CI, conformity index; DC, Dice coefficient; NTCP, normal tissue complication probability.
